# On the occurrence of intracolonial genotypic variability in highly clonal populations of the hydrocoral *Millepora platyphylla* at Moorea (French Polynesia)

**DOI:** 10.1038/s41598-017-14684-3

**Published:** 2017-11-01

**Authors:** Caroline E. Dubé, Serge Planes, Yuxiang Zhou, Véronique Berteaux-Lecellier, Emilie Boissin

**Affiliations:** 1 PSL Research University: EPHE-UPVD-CNRS, USR 3278 CRIOBE, Université de Perpignan, 52 Avenue Paul Alduy, 66860 Perpignan Cedex, France; 2 Laboratoire d’Excellence “CORAIL”, USR 3278 CRIOBE, BP 1013, 98729 Papetoai Moorea, French Polynesia; 3 UMR 250/9220 ENTROPIE, IRD-UR-CNRS, LabEx “CORAIL”, 101 Promenade Roger-Laroque, BP A5, 98848 Nouméa New-Caledonia, France

## Abstract

Intracolonial genotypic variability is described in many colonial organisms and arises from mosaicism (somatic mutation) and/or chimerism (allogenic fusion). Both processes provide an additional source of genotypic variation in natural populations and raise questions on the biological significance of colonies having more than one genotype. Using fifteen microsatellite markers, we screened for potential genetic heterogeneity within *Millepora platyphylla* colonies, a hydrocoral species known for its extensive morphological plasticity among reef habitats. We aimed to determine whether mosaicism and chimerism were related to specific reef habitats and/or colony morphologies. Our results show that intracolonial genotypic variability was common (31.4%) in *M. platyphylla* at Moorea, French Polynesia, with important variations in its frequency among habitats (0–60%), while no effect of morphology was observed. Mosaicism seemed responsible for most of the genetic heterogeneity (87.5%), while chimerism was rarer. Some mosaics were shared among fire coral clones indicating that mutations could be spread via colony fragmentation. Further, the genotypic variability among clones suggests that colonies produced asexually through fragmentation have the potential to accumulate their own mutations over time. Such mutation dynamics might have important implications for the adaptive potential of long-lived reef-builder populations that are predominantly sustained through asexual reproduction.

## Introduction

Understanding evolutionary strategies in species largely relies on the concept of individuality, where each individual represents the unit on which selection pressures occur^1^. An individual is intrinsically defined as reproductive, physiologically autonomous, genetically unique and homogeneous^2,3^, but there are many studies that question this definition^4^. For instance, it has been notably recognised that for colonies of social insects, physiological unity is not respected because individuals cooperate with others to form a “superorganism”, acting as though the colony was one single individual^5^. Asexual reproduction is also common in natural populations of countless plants and animals, where individuals are not genetically unique^6^. At last, the occurrence of genetic heterogeneity within a single individual has been documented in populations of protists, fungi, plants and animals^4^ and is now considered a common phenomenon.

There are two main processes that can lead to intra-individual genotypic variability in natural systems: mosaicism and chimerism. Mosaicism is the outcome of intrinsic genetic changes within a single colony caused, among other processes, by somatic mutations^7^. In contrast, chimerism originates from the fusion of at least two individuals of the same species (allogenic fusion) and requires specific environmental conditions and species’ life history traits^8^. Based on such definitions, chimerism is expected to generate a greater genetic variation within the successfully merged colony compared to mosaicism. Chimerism is generally rarer than mosaicism in natural populations due to restricting allorecognition systems^4^. This process mostly occurs in seaweeds and colonial marine organisms with a dispersive pelagic phase, such as sponges, hydroids, bryozoans, ascidians and corals^7,9–11^. Mosaicism, by contrast, is a widespread mechanism of many clonal plants and animals with long life-spans^12–14^.

Chimeras and mosaics may confer both benefits and disadvantages at the individual level. Theoretically, intra-individual genotypic variability may promote disruptive internal conflicts threatening an organism’s ability to function, such as developmental instability^15,16^ and intra-individual competition^17,18^. However, the co-occurrence of many different genotypes within an individual also generates additional genotypic variation in natural populations. Such variability may result in more versatile phenotypic traits (e.g. physiological pathways and morphologies), which can further increase the potential for adaptation^19–21^ through intra-individual selection pressures (e.g. growth rate, reproductive success and survivorship)^4,14,22,23^. Even so, this additional genotypic diversity will only affect the fitness of an individual and not its population, unless it is possible for the genetic variation to be passed on to the gametes. Individuals having more than one genotype may thus facilitate the adaptive potential of long-lived organisms that reproduce primarily through asexual reproduction. As both chimerism and mosaicism generate intra-individual genotypic variation, evaluating the occurrence of these processes in threatened species such as reef-building corals may carry important implications for their conservation.

Many studies have shown the occurrence of intracolonial genotypic variability in scleractinian corals^24–29^. Due to the existence of multipotent stem cells (MPSCs) in colonial reef organisms^30^, the propagation of somatic mutations within a colony is likely to occur^14^ when mutations are not detrimental. These mutations are potentially passed on to the next generation of gametes due to the capacity of MPSCs to generate germline stem cells (GSCs)^28,31^. In partially clonal reef organisms, somatic mutations can also be spread by means of asexual reproduction pathways, which include fragmentation, budding, polyp bail-out, asexually produced planula and embryo breakage^32,33^.

Our understanding of intracolonial genotypic variability in colonial reef species (e.g. soft corals^34^, sponges^35^ and scleractinian corals^24–29,36^) has improved over the last decade. Until very recently, such information was unavailable for *Millepora* hydrocorals^37^ (‘fire corals’) despite their major contribution to the reef framework in some reef ecosystems^38^. *Millepora* species inhabit a wide range of habitats^39,40^ and often grow into large colonies that pre-empt space and compete with scleractinian corals^38,41^. Fire corals alternatively reproduce by shifting from asexual pathway of fragmentation to sexual reproduction via gonochoric broadcasting of both medusoids and planula larvae^39,42^. The simultaneous use of sexual/asexual reproductive modes has been recorded in *Millepora platyphylla* at Moorea, French Polynesia, where habitat specific environmental conditions are thought to determine the levels of clonality and intraspecific variations in colony morphology^43^. Such variability in life history traits leads to marked differences in population structure^40^ and genotypic diversity among reef habitats^43^. However, the sets of the biological and environmental conditions influencing intraorganismal genotypic variability are still ambiguous.

**Figure 1:**
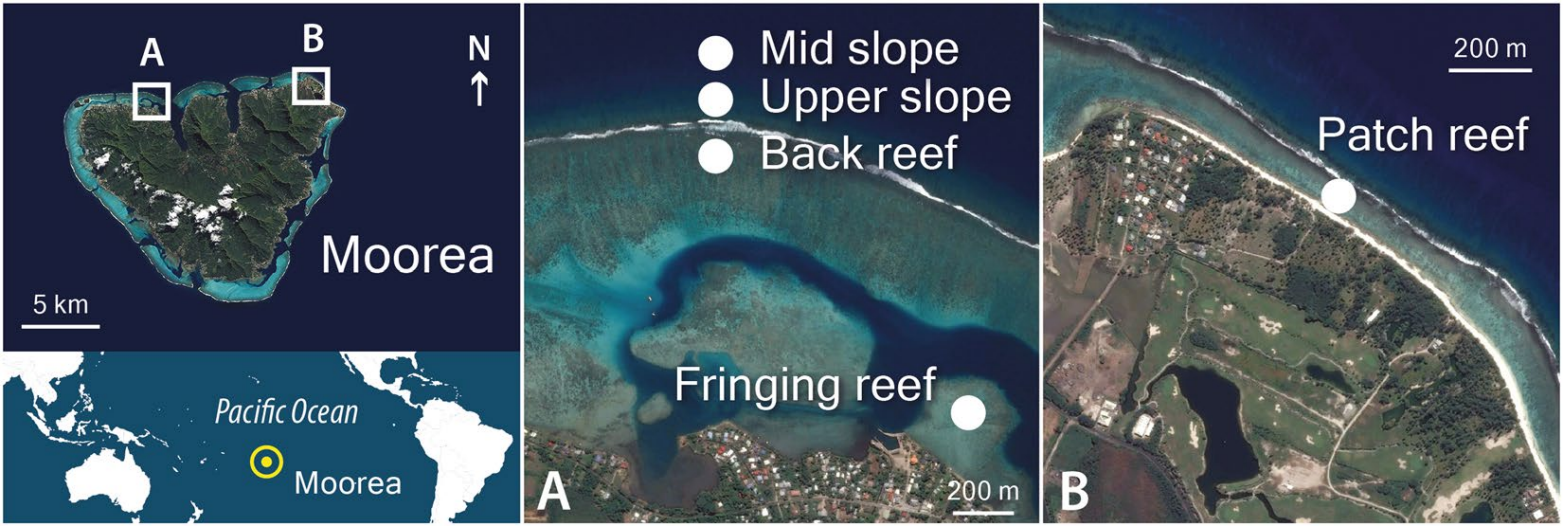
Aerial views of the five habitats surveyed in Moorea, French Polynesia. The following are the names of the surveyed site: (**A**) Papetoai and (**B**) Temae. The world map was obtained from Aix-Marseille University (http://www.d-maps.com) and images from Google Earth (Map data © 2015 Google, DigitalGlobe). The figure was created using Adobe Photoshop CS6 software.

Using fifteen microsatellite markers, we screened for potential intracolonial genotypic variability within populations of the hydrocoral *M. platyphylla* in five reef habitats in Moorea (French Polynesia); two on the fore reef: mid slope and upper slope, and three in the lagoon: back reef, fringing reef and patch reef (Fig. 1). We specifically addressed the following questions: how common is intracolonial genotypic variability in the fire coral *M. platyphylla*? What is the major process, i.e. mosaicism or chimerism, leading to genetically heterogeneous colonies? Is the level of intracolonial genotypic variability related to a specific habitat and/or coral morphology?

## Methods

### Sampling

Between April and December 2013 field surveys were conducted on the north shore of Moorea, French Polynesia, at three different locations (Tiahura, Papetoai and Temae) across five reef habitats; two in the fore reef: mid slope (13 m depth) and upper slope (6 m depth), and three in the lagoon (<1 m depth): back reef, fringing reef and patch reef (Fig. 1). From these surveys a total of 51 colonies of *M. platyphylla* were collected (CITES – FR1298700028–E) to test for intracolonial genetic variability in fire corals (Table 1). All colonies were subjected to a multiple sampling design, where five tissue samples were collected from each colony with four samples taken from the edges of the colony (cardinal points) and one from the centre of the colony. This sampling strategy was previously used to detect genetically heterogeneous individuals in some coral^29^ and hydrocoral species^37^. Selected colonies had a minimum size of 500 cm² to ensure sexual maturity and showed no visual evidence of fusion between two or more individuals (i.e. various morphologies and colours within a single colony, and no interaction zone). To determine whether the colony morphology influences the prevalence of intracolonial genetic variability, we classified each colony in one of the following morphologies: 1) massive: solid colonies, roughly hemispherical in shape, 2) encrusting: thin colonies growing against the substratum or 3) sheet tree: encrusting bases with vertical bladelike outgrowths^40^. The size of each colony (standardised as the projected surface in cm²) was estimated from 2D photographs using ImageJ 1.4 f software^44^. Photographs were taken from above the colony and included a plate of known dimensions positioned next to each colony. Overall, a total of 255 small fragments (51 colonies × 5 samples) of tissue-covered skeleton (<2 cm^3^) were collected and preserved in 80% ethanol and stored at −20 °C until DNA extraction. Field experiments were approved by the Presidency of French Polynesia (#0085) and performed in accordance with relevant Polynesian regulations.

**Table 1:** Sampling pattern among the five surveyed habitats.

Habitat	Depth (m)	# Colonies	# Samples	# Fragments	# MLGs	# Clonal MLGs	# Clones	# Hetero colonies	Morphology (N)			Colony size
									MA	EN	ST	± SE (cm²)
Patch	0.82	10	5	50	17	1	2	6	10	—	—	11 272 ± 8 822
Fringing	0.81	10	5	50	12	1	2	2	10	—	—	8 976 ± 4 494
Back	0.74	8	5	40	7	1	2	0	2	—	6	4 289 ± 3 111
Upper	5.90	13	5	65	17	1	3	6	—	2	11	29 749 ± 23 811
Mid	12.92	10	5	50	12	0	0	2	3	4	3	18 459 ± 11 107
Total	—	51	—	255	65	4	9	16	25	6	20	15 536 ± 15 917

^#^Colonies, number of colonies sampled; ^#^Samples, number of samples within a single colony; ^#^Fragments, total number of tissue-covered skeleton collected; ^#^MLGs, number of detected multilocus genotypes; ^#^Clonal MLGs, number of repeated multilocus genotypes (clonal genotypes); ^#^Clones, number of clone mates; ^#^Hetero colonies, number of genetically heterogeneous colonies; Morphology, number of colonies with a massive (MA), encrusting (EN) or sheet tree (ST) morphology and mean colony sizes are given for the 51 colonies sampled and ± SE for variation among colonies.

### Microsatellite genotyping

Samples were incubated at 55 °C for 1 hr in 450 µL of digest buffer with proteinase K (QIAGEN, Hilden, Germany). Genomic DNA was extracted using a QIAxtractor automated genomic DNA extraction instrument, according to manufacturer’s instructions. Samples were amplified and genotyped at fifteen microsatellite loci shown to be coral-specific and polymorphic in *M. platyphylla* ^45,46^ (see Supplementary Table 1 for more details). All loci were combined in three multiplex panels according to their size range and primer annealing temperature. PCRs were performed in a final volume of 10 µL including 5 µL Type-it Multiplex PCR Master Mix (1x) (QIAGEN, Hilden, Germany), 3 µL RNase-free water, 1 µL primers (2 µM of fluorescently labelled forward primer – G5 dye set including 6-FAM, VIC, NED and PET – and reverse primer diluted in TE buffer) and 1 µL of template (10 to 50 ng.µL^−1^). The PCR protocol included an initial denaturing step of 5 min at 95 °C, followed by 40 cycles of 30 sec at 95 °C, 90 sec at 57–63 °C, and 30 sec at 72 °C, and by a final 30 min elongation step at 60 °C. PCR products were sent to GenoScreen platform (Lille, France) for fragment analysis and were visualised using an Applied Biosystems 3730 Sequencer. An internal size ladder (GeneScan 500 LIZ, Applied Biosystems) was used for accurate sizing and alleles were scored and checked manually using GENEMAPPER v.4.0 (Applied Biosystems, Foster City, CA). Samples that were ambiguous in their scoring were re-amplified and re-scored, as for missing alleles. Alleles were individually re-scored by a second and third person to ensure accurate genotyping. All peak profiles that were faint or ambiguous (i.e. multiple peaks) were considered as missing data and only samples with no more than two missing loci were retained for further genetic analyses.

### Data analyses

Controls for the presence of null alleles and large allele dropout were performed with MICRO-CHECKER v.3.7^47^. Multilocus genotypes (MLGs) were produced for each sample and compared within each colony to detect the occurrence of intracolonial genotypic variability. The genotype probability (GP) was estimated for each locus and for a combination of all loci using GENALEX v.6.5^48^. Repeated multilocus genotypes were also identified in GENALEX and were considered as clone mates at GP < 0.001 (Table 1). For the 51 sampled colonies of *M. platyphylla* that were subjected to multiple sampling, the most common genotype was retained as the main genotype. All additional genotypes within the same colony could result either from mosaicism (somatic mutations) or chimerism (fusion of two or more individuals). In previous studies, mosaic individuals were identified based on the number of divergent loci from the main genotype, i.e. only one or two loci (as in^26,28^) since mutations remain rare events^49^. In contrast, a greater number of loci and allelic differences were expected in chimeras, i.e. when two genetically distinct colonies merge. Based on the stepwise mutation model of microsatellite markers^50^, we estimated the number of repeat units that were added or subtracted during a mutation event. Divergent alleles from the main genotype caused by multiple-step mutations and large allele differences are most likely due to chimerism rather than somatic mutations. Stepwise mutations were identified over all loci for each deviating genotype and averaged per habitat and morphology (percentage of stepwise mutations).

Bayesian clustering analyses have been used to identify chimeras based on their cluster assignment probability. Chimeras have to include genotypes that differ from the main genotype and belong to a different cluster^27,29^. Here, mosaic individuals and chimeras were identified based on a Bayesian clustering analysis using STRUCTURE^51^. Clustering analyses were performed to ensure non-biased detection of deviating genotypes following the protocol used in Schweinsberg *et al*.^29^. Initial STRUCTURE runs were used to determine the most likely number of clusters (K) in each population of *M. platyphylla*, i.e. within the five reef habitats: mid slope, upper slope, back reef, fringing reef and patch reef. Runs were performed with the default setting, a burn-in period of 50 000, 50 000 MCMC repeats and 10 iterations per K. The results were uploaded to STRUCTURE HARVESTER^52^ and the most likely K was retained for a second run in STRUCTURE with a burn-in period of 500 000, 500 000 MCMC repeats, 10 iterations and uniform prior setting. The results were once again uploaded to STRUCTURE HARVESTER and the resulting merged dataset was analysed to estimate cluster assignment. Based on our definitions of chimeras and mosaics using microsatellite data, we assumed that only fragments of the same colony having a genetic variation of at least 60%, whatever the number of divergent loci, were the result of chimerism^29^. All other deviating genotypes were considered as mosaic colonies.

### Statistical analyses

Chi square tests with Monte Carlo simulation (1000 replicates) were used to assess for differences in the relative numbers of colonies that harbour single genotypes, somatic mutations and chimeras among the five reef habitats and three colony morphologies. Differences in stepwise mutations (one-step, two-step, three-step and four- to twelve-step) among habitats, morphologies and between mosaic colonies and chimeras were also tested using Chi square tests with Monte Carlo simulation. Pearson’s correlation coefficient was used to determine whether the occurrence of intracolonial variability increased with the colony size. All statistical analyses were performed in the R programming environment v2.15.1^53^.

## Results

### Genotypic diversity and morphology

Out of the 255 samples collected from 51 colonies of the hydrocoral *M. platyphylla*, 65 multilocus genotypes were identified (Table 1). Clone mates were detected in almost all reef habitats and only two colonies shared the same genotype in the patch reef, fringing reef and back reef, and three colonies in the upper slope. All colonies sampled in the mid slope were genetically unique (Table 1). The sheet tree morphology was dominant in the upper slope (85%) and back reef (75%), while all colonies were massive in nearshore habitats (fringing and patch reefs). The growth form of fire coral colonies was highly variable in the mid slope, where the three morphologies were found in equal proportions (Table 1). Although the size varied greatly among colonies and habitats, colonies of fire corals were smaller in the back reef (4,289 cm^2^) compared to other habitats (8,976–29,749 cm^2^, Table 1).

### Identification of intracolonial genotypic variability

**Figure 2:**
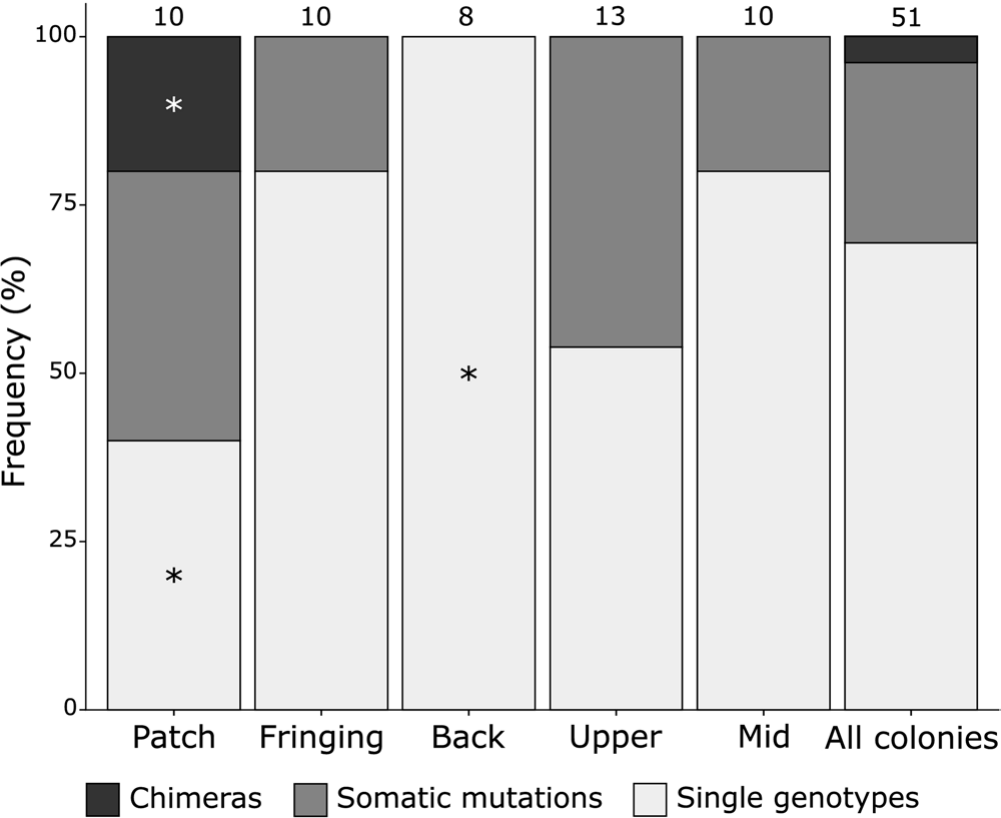
Intracolonial genotypic variability detected in *M. platyphylla* colonies in the five surveyed habitats. Relative numbers of colonies (frequencies in percentages) harbouring one or many genotypes. Colonies harbouring a single genotype are shown in light grey; colonies with multiple genotypes caused by mosaicism (somatic mutation) are shown in grey and by chimerism (allogenic fusion) in black. Numbers above each set of bars are the total number of colonies and stars indicate statistical difference for a given intracolonial genetic stage among habitats (P < 0.05).

**Figure 3:**
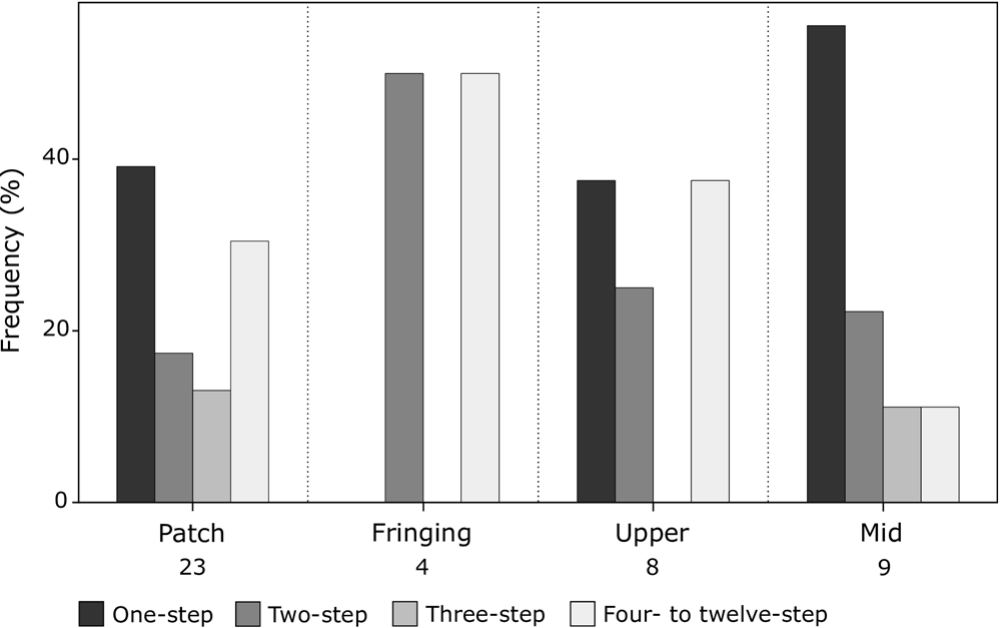
Frequency (%) of deviating genotypes caused by one-step to four- to twelve-step mutations over all loci in all surveyed habitats. Numbers below each set of bars are the total number of deviating genotypes. Notice that the back reef is not shown since no deviating genotype was found within this habitat and there was no statistical difference for step mutation pattern among habitats.

Among the 51 tested colonies of fire corals, 16 (31.4%) harboured more than one single genotype (Fig. 2 and Table 1). First, it should be noted that the number of heterogeneous colonies does not increase with the colony size (r = 0.45, *P* = 0.44). The occurrence of intracolonial genotypic variability differed significantly among the five surveyed habitats (Chi square test, *P* < 0.05, Fig. 2), while no difference was found among the three morphologies. The percentages of genetically heterogeneous colonies were highest in the patch reef (60.0%) and upper slope (46.2%), followed by the fringing reef and mid slope (20.0% each), and finally the back reef, where all colonies were genetically homogeneous (Fig. 2 and Table 1). In the patch reef and upper slope, where heterogeneous colonies are more common, nearly 70% of the deviating genotypes were caused by one-step and four- to twelve-step mutations (Fig. 3). Two-step and four- to twelve-step mutations contributed equally in creating divergent genotypes in the fringing reef, while deviating genotypes were mostly caused by one-step mutations in the mid slope (Fig. 3). Regardless, no significant difference was found for the stepwise mutation pattern among reef habitats and colony morphologies.

### Clustering analyses: mosaicism versus chimerism

**Figure 4:**
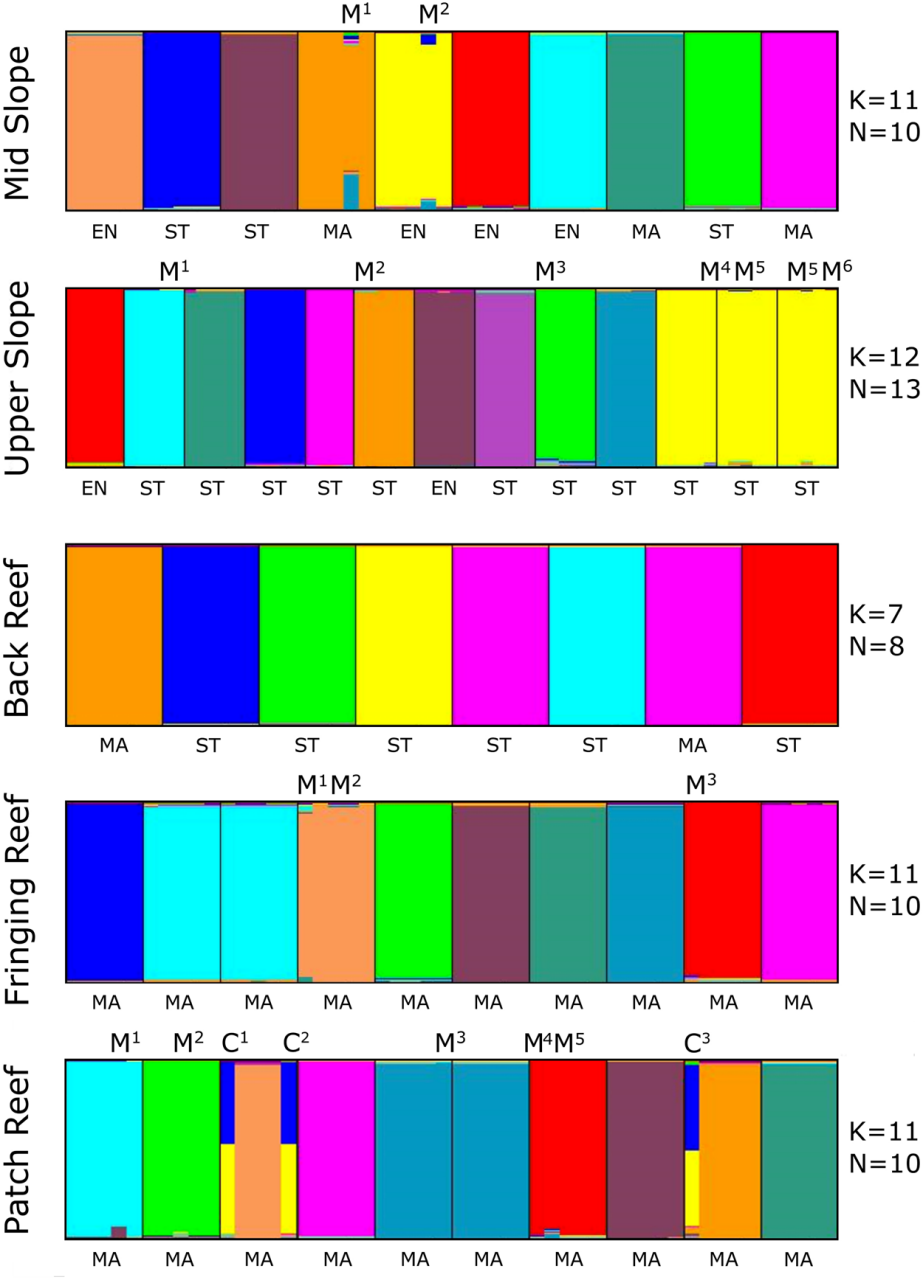
Assignment analyses based on Bayesian clustering showing mosaic colonies and chimeras in the five surveyed habitats. Bar plot for N colonies and K clusters are shown per habitat. The x-axes show colony identification and whether they display a massive (MA), encrusting (EN) or sheet tree (ST) morphology, and y-axes show the cluster membership. Samples marked with M show deviating genotypes due to mosaicism, C are chimeras, and each number associated with M and C represents one deviating genotype. Colonies with the same colour within each of the five bar plots depict clone mates.

Based on our definitions of mosaicism and chimerism, 14 mosaic colonies and 2 chimeras were identified in total among the 51 colonies of *M. platyphylla* screened (Fig. 4). The significant highest percentage of heterogeneous colonies (60%) was found in the patch reef with four mosaics and two chimeras (Chi square test, *P* < 0.05, Fig. 2). In this latter habitat, mosaic colonies differed from the main genotype with a maximum of two loci (Supplementary Table 2) and one of the mosaics harboured multiple genotypes, i.e. more than two genotypes (patch reef bar plot, colony No. 7, Fig. 4). Genotypic variability was also detected among colonies sharing the same genotype (clones). One of the two clones identified in the patch reef was genetically homogenous (colony No. 6), while the other had one deviating genotype due to somatic mutations (colony No. 5, Fig. 4). Chimeras were detected in two colonies within the patch reef. The colony No. 3 had two deviant genotypes: C^1^ with ten divergent loci and C^2^ with nine (Fig. 4 and Supplementary Table 2). Allelic variations in both of these divergent genotypes were caused by one- to four-step mutations (Supplementary Table 2). The colony No. 9 had only one deviant genotype, C^3^ with nine divergent loci due to one- to twelve-step mutations (Fig. 4 and Supplementary Table 2). In the fringing reef, two mosaic colonies were identified; one displayed multiple genotypes (colony No. 4), and the other had only one divergent allele (colony No. 9, Fig. 4 and Supplementary Table 2). In the upper slope, three of the six deviating genotypes were detected within the three identified clones. One clone (colony No. 13, the smallest clone; see Supplementary Table 2) displayed multiple genotypes: where one deviating genotype differed by only one allele from the main genotype (M^6^) and a second one (M^5^) had four divergent loci (mostly from one- and two-step mutations) and was shared with one of its clone mates (colony No. 12, Fig. 4). A two-step mutation in the highly divergent genotype (M^5^) resulted in another deviating genotype in the third clone (M^4^, colonies No. 11). In the mid slope, the two mosaic colonies differed by five to six loci from the main genotype mostly due to one- to three-step mutations.

Overall, there was no significant difference for the stepwise mutation pattern between identified chimeras and mosaic colonies. At last, there was no difference in the relative numbers of colonies harbouring single genotypes, somatic mutations and chimeras among the three morphologies. Mosaic colonies were identified in all *M. platyphylla* growth forms with most of the somatic mutations occurring in massive (7 out of 14) and sheet tree morphologies (6 out of 14, Fig. 4).

## Discussion

This study demonstrates that the occurrence of intracolonial genotypic variability in *M. platyphylla* is common at Moorea (31.4%), while important variations in its frequency were found among habitats. Our results reveal that chimerism is restricted to the patch reef habitat and thus seemed rarer compared to mosaicism. Even so, the fusion between siblings may increase opportunities for the development of chimeras in fire corals. Furthermore, the genetic dissimilarity among clones revealed that asexual reproduction affects the prevalence of mosaic colonies in fire coral populations. Somatic mutations that arise within a colony can be spread in the population via its fragmentation and asexual fragments produced from the same mother colony have the ability to accumulate their own mutations over time. Such propagation and accumulation of somatic mutations may represent an efficient means to increase the genetic diversity in *M. platyphylla* populations at Moorea, which are predominantly sustained through asexual reproduction^43^.

### Intracolonial genotypic variability in *M. platyphylla*

The occurrence of intracolonial genotypic diversity was traditionally assumed to be a phenomenon of rare exception^7,18^. However, recent investigations have demonstrated that genotypic heterogeneity is widespread in scleractinian corals^24–29^. Our results show that intracolonial genotypic diversity is also common in hydrozoans, such as the fire coral *M. platyphylla*, with important variations in its frequency among reef habitats. Our genotypic data from 51 colonies (with five replicates sampled per colony) revealed a high proportion of genetically heterogeneous colonies (31.4%). Such occurrence of intracolonial genotypic variability corroborates recent observations in branching *Acropora* corals (38.7%, in^29^), but is much higher when compared to another branching coral, *Seriatopora hystrix* (17.4%, in^27^). A recent study based on limited sampling (10 colonies) and less molecular markers (6 microsatellite loci) has demonstrated that intracolonial genotypic variability was a rare phenomenon in *M. platyphylla* at Moorea (10%)^37^. Here, our panel of fifteen microsatellite loci showed that populations of *M. platyphylla* can display up to 60% of genetically heterogeneous colonies, which is much higher than what was previously observed in both scleractinian and hydrozoan corals^27,29,37^. Increasing the number of loci and colonies screened most likely increases the discriminative power to detect intracolonial genotypic variability in natural populations, a phenomenon that may have been underestimated in previous studies. However, it has to be noted that our definitions of mosaicism and chimerism varies from some other studies. Some have compared allelic differences within a heterogeneous colony to the one observed at the population level, i.e. allelic differences expected from sexual reproduction^27^. This approach is less conservative compared to the one we used and yet our proportions of heterogeneous colonies were higher. When compared to other studies with the same approach that we used^29,37^, the occurrence of intracolonial genotypic is similar or higher to the ones recorded in scleractinians and hydrocorals.

### Intracolonial genotypic variability among reef habitats

Fire corals were sampled in five different reef habitats where colonies were exposed to different environmental conditions. The variability of environmental settings can potentially influence the rate of somatic mutational divergence within a species^54^. Colonies were collected at different depths leading to varying exposure to UV-induced DNA damage^55^, which often derives in somatic mutations^56^. Variations in the proportions of heterogeneous colonies among the five surveyed habitats (0–60%) seemed unrelated to differences in their exposure to solar radiation. In Moorea, fire corals are exposed to high solar irradiance in the back and fringing reefs^57^. However, both of these shallow habitats (<1 m depth) were characterised by low proportions of heterogeneous colonies (0% and 20%, respectively) and were similar to those of the mid slope population (20%) where irradiance is lower. Rather than UV radiation variability, the occurrence of intracolonial genotypic variability in *M. platyphylla* might be related to habitat specific life history strategies. Populations of fire corals can evolve differences in reproductive and morphological traits among contrasting reef habitats^43^, which may further influence the opportunity for chimerism and mosaicism to occur^14,28,36^.

### Chimerism

A low proportion of chimeras was identified in the fire coral population (2 out of 51 colonies sampled, i.e. 3.9%), which is similar to earlier reports for scleractinian corals (from 1.3 to 4.5% depending on the coral species, e.g.^27,29^). This result further suggests that chimeric fusions between conspecific are rare events in dynamic environments such as coral reefs. Two chimeras were detected in the patch reef habitat, where *M. platyphylla* primarily reproduce through fragmentation rather than sexual reproduction^43^. In Moorea, fusion between siblings is likely to occur as fire corals have limited dispersal abilities and are often aggregated due to the co-settlement of their larvae (Dubé, unpubl. data). Puill-Stephan *et al*.^58^ demonstrated that high levels of relatedness between juvenile corals correlated with late maturation of allorecognition. The fusion of siblings could thus be related to a low conspecific acceptance threshold and/or a delay in allorecognition maturation for *Millepora* hydrocorals, as described in some hermatypic corals^24,26^. Such delay in the recognition system can increase opportunities for chimeric fusion between adjacent recruits (i.e. early life stages), which seems to offer advantages. Advantages include the establishment of a colony with an increased genetic repertoire and a reduced onset of reproduction, increased competitive capabilities and growth during early development and reduced colony mortality^4,24,26,59–61^. While enhancing intracolonial genetic heterogeneity, chimerism may result in different expressed phenotypes that might each withstand a different set of environmental pressures.

### Mosaicism

Fourteen adult colonies exhibited genotypic variability due to mosaicism. This indicates that the accumulation of somatic mutations might well be a common phenomenon in *M. platyphylla*. Mosaic colonies were found in all reef habitats except for the back reef. The incidence of genetic mosaicism is expected to increase with age and size due to a higher number of dividing cells available for mutation^49^. Therefore, the absence of heterogeneous colonies in the back reef could be related to higher mortality of larger colonies^40^, hence limiting the colony size (i.e. growth and age). This limitation in size will further lower the accumulation of mutations in adult corals. Nevertheless, mosaicism was found in colonies of various sizes (from 800 to 63,500 cm^2^), with some of the largest colonies being genetically homogeneous (at least from the five samples analysed for each colony). In partially clonal organisms, such variability in the accumulation of mutations among size classes can be related to small recently fragmented clones that might have accumulated mutations before their fragmentation. For *Millepora* hydrocorals, clones are most likely produced by colony fragmentation as the production of asexual larvae has never been described so far for this genus. Furthermore, as suggested by our results, clones are potentially produced through different fragmentation events (multiple generations). Such a life history strategy highlights the importance of aging clones within genetic lineages (as described in^62^) rather than estimating colony size to better understand the mechanisms behind mosaicism in reef-building corals.

*M. platyphylla* is also morphologically variable and can have massive, encrusting or sheet tree morphotypes. In this study, mosaicism was identified in all *M. platyphylla* growth forms, but mostly in the massive and sheet tree ones. In many colonial organisms, such as long-lived trees and corals, branching growth forms usually exhibit more deviating genotypes throughout the entire colony due to mutations that only occur within isolated branches^14,63^. On the contrary, the propagation of somatic mutations is less likely in massive growth forms because polyps are in close contact with one another. Such an interaction favours intracolonial competition, which often results in the elimination of alternative mutant cells^17,18^. In the upper slope, most of the colonies grew as isolated vertical blades on encrusting bases (i.e. sheet tree), which is similar to the branching morphology. Consequently, somatic mutations would be expected to be more abundant in sheet tree colonies. However, half of the mosaic colonies were observed within massive colonies and raise questions on whether colony morphology influences mosaicism processes. Schweinsberg *et al*.^29^ also demonstrated a conflicting pattern of mosaicism among coral growth forms, whereby the highest and lowest proportions of mosaic individuals were both detected in branching species. Whether more extensive studies could verify an increased accumulation of somatic mutations in branching corals (or tubular or sheet) compared to massive or encrusting growth forms remains to be determined.

Despite our random sampling scheme, clone mates (colonies produced through fragmentation and assumed to be genetically identical) were collected in almost all reef habitats with the exception of the mid slope. The absence of fire coral clones in this latter habitat must be related to the high investment in sexual reproduction reported at Moorea, where clones were less abundant^43^. Furthermore, the morphological plasticity of fire corals has been reported to highly influence the level of clonality among reef habitats at Moorea due to differences in growth form’s sensitivity to fragmentation^43^. Regardless, there was no effect of the morphology on the occurrence of mosaicism within a colony. However, three clone mates were identified in the upper slope and were comprised of four different genotypes. Some genotypes were shared among asexual fragments (clones produced through fragmentation) and others were found only in one of the three fragments. This result indicates that when fire corals spread via colony fragmentation, the fragments inherit somatic mutations from their mother colony, in addition to acquiring their own over time. Hence, clonal reproduction can often result in mosaic colonies having deviating genotypes of more than four divergent loci, although mosaicism was commonly thought to induce divergent genotypes at one or two loci^49^. Furthermore, it is also possible that mosaic colonies result from multi-step mutations in long-lived organisms, such as fire corals. Consequently, there was no difference in the stepwise mutation pattern among heterogeneous colonies that were produced through mosaicism or chimerism, although this latter process was thought to induce more mutation steps within the merged colony. Considering the common occurrence of mosaicism in *M. platyphylla*, this process might have important implications for its population genetic variation.

### Evolutionary and ecological implications

Overall, our study reveals that mosaicism is a very promising process to increase genotypic variability in *M. platyphylla*, a species that mostly relies on colony fragmentation for colonisation and population persistence^43^. Our microsatellite data showed that clonal genets can accumulate allelic mutations that can be spread in the population via asexual reproduction. However, further investigations are needed to ensure that there is no barrier to prevent somatic mutations of stem cells from being spread in the population via the next sexual generation in *M. platyphylla*. More studies on the occurrence of mutations occurring in coding and/or regulatory regions of the genome are also needed to examine whether such genetic variation become functionally variable under strong selection pressures. Such mutation dynamics can potentially result in more versatile phenotypic traits and facilitate adaptation processes of partially clonal organisms^64–66^. Nevertheless, the high level of standing genetic variation that results from sexual recombination in *M. platyphylla* ^43^ might as well be far greater and thus more influential on adaptation of fire coral populations than somatic mutations. Even if chimerism is less common in the studied population, this process can confer various ecological advantages and can further benefit adaptation. Regardless of the intrinsic costs incurred with chimerism, such as cell competition and parasitism^17,18^, chimeras have the ability to present their best-fitted genetic combination in response to environmental changes^24^. It is thus imperative to expand studies of intracolonial genotypic variability based on neutral microsatellite markers and to further include functional genes that underpin coral physiology, which often correlates with adaptive advantages.

## Electronic supplementary material

Supplementary Tables
