# Supplementary material for: On the occurrence of intracolonial genotypic variability in highly clonal populations of the hydrocoral Millepora platyphylla at Moorea (French Polynesia)
Source: Sci Rep. 2017 Nov 1;7:14861. doi: 10.1038/s41598-017-14684-3 (PMC5665921; doi:10.1038/s41598-017-14684-3)
Supplement: Supplementary file 1 — Supplementary Tables [file 41598_2017_14684_MOESM1_ESM.pdf]

On the occurrence of intracolonial genotypic variability in highly clonal populations of the hydrocoral *Millepora platyphylla* at Moorea (French Polynesia)

Caroline E. Dubé*, Serge Planes, Yuxiang Zhou, Véronique Berteaux-Lecellier, Emilie Boissin

*Correspondence: caroline.dube.qc@gmail.com

Supplementary Information

Table S1 – Summary of genetic diversity at each of the fifteen microsatellite loci in *M. platyphylla.*

Table S2 – Summary of colony/fragment identification, colony morphology, colony size and genotypic data for each fragment of *M. platyphylla* at the fifteen microsatellite loci.

Table S1. Summary of genetic diversity at each of the fifteen microsatellite loci in *M. platyphylla.*

Locus	Motif	T_A (°C)	MP	Na	Size range (bp)	Developed by
Mill07	$(CA)_{16}$	57	3	18	92–130	Dubé *et al.* 2017
Mill27	$(TG)_{10}$	57	3	5	140–148	Dubé *et al.* 2017
Mill30	$(TG)_{11}$	57	2	4	203–211	Dubé *et al.* 2017
Mill47	$(GA)_{8}$	57	2	4	114–124	Dubé *et al.* 2017
Mill52	$(AC)_{9}$	63	1	3	94–98	Dubé *et al.* 2017
Mill67	$(TAGA)_{6}$	63	1	6	275–345	Dubé *et al.* 2017
Mill93	$(TGT)_{7}$	57	2	2	94–100	Dubé *et al.* 2017
Mill94	$(GAA)_{7}$	57	3	4	134–143	Dubé *et al.* 2017
Mill95	$(TTG)_{7}$	63	1	4	123–138	Dubé *et al.* 2017
Mill101	$(CAA)_{6}$	57	2	2	132–135	Dubé *et al.* 2017
Mill103	$(AG)_{7}$	57	3	6	94–114	Dubé *et al.* 2017
Mill_D01	$(ACCG)_{9}$ $(ACTG)_{3}$	57	2	4	169–197	Heckenhauer *et al.* 2014
Mill_D04	$(AAAT)_{6}$	57	2	4	152–166	Heckenhauer *et al.* 2014
Mill_D06	$(AAT)_{9}$	63	1	13	136–187	Heckenhauer *et al.* 2014
Mill_D08	$(GAT)_{11}$	63	1	3	97–118	Heckenhauer *et al.* 2014

TA, Annealing temperature (°C); MP, multiplex panel in which each locus was included; Na, Number of alleles per locus.

Supplementary references

Dubé, C. E., Planes, S., Zhou, Y., Berteaux-Lecellier, V. & Boissin, E. Genetic diversity and differentiation in reef-building *Millepora* species, as revealed by cross-species amplification of fifteen novel microsatellite loci. *PeerJ* **5,** e2936 (2017).

Heckenhauer, J. *et al*. Isolation, characterization and cross amplification of eleven novel microsatellite loci for the hydrozoan coral *Millepora*. *Conserv. Genet. Resour.* **7,** 215–217 (2014).

Table S2. Summary of colony/fragment identification, colony morphology, colony size and genotypic data for each fragment of *M. platyphylla* at the fifteen microsatellite loci.

ID Col	ID Frag	Mor	Size	Mill52	Mill67	Mill93	Mill95	MillD06	MillD08	Mill30	Mill47	Mill101	MillD01	MillD04	Mill07	Mill27	Mill94	Mill103
PR 1	PR1-F1	MA	12064	9494	275275	9494	123129	136175	9797	205205	114114	132132	169193	162166	127127	143145	134137	94100
PR 1	PR1-F2	MA	12064	9494	275275	9494	123129	136175	9797	205205	114114	132132	169193	162166	127127	143145	134137	94100
PR 1	PR1-F3	MA	12064	9494	275275	9494	123129	136175	9797	205205	114114	132132	169193	162166	127127	143145	134137	94100
PR 1	PR1-F4	MA	12064	9494	275275	9494	123129	136175	9797	205205	114114	132132	169193	162166	125125	143145	134137	94100
PR 1	PR1-F5	MA	12064	9494	275275	9494	123129	136175	9797	205205	114114	132132	169193	162166	127127	143145	134137	94100
PR 2	PR2-F1	MA	19007	9494	307307	9494	123123	142175	9797	207211	118118	135135	169193	162166	113123	143145	134134	9494
PR 2	PR2-F2	MA	19007	9494	307307	9494	123123	142175	9797	207211	118118	135135	169193	162166	113123	143145	134134	9494
PR 2	PR2-F3	MA	19007	9494	307307	9494	123123	142175	9797	207211	118118	135135	169193	162166	113123	143145	134134	94100
PR 2	PR2-F4	MA	19007	9494	307307	9494	123123	142175	9797	207211	118118	135135	169193	162166	113123	143145	134134	9494
PR 2	PR2-F5	MA	19007	9494	307307	9494	123123	142175	9797	207211	118118	135135	169193	162166	113123	143145	134134	9494
PR 3	PR3-F1	MA	26997	9698	275275	9494	123123	136187	100100	205207	120120	132135	169193	158166	111111	143145	134137	9698
PR 3	PR3-F2	MA	26997	9494	275275	9494	123123	142175	9797	207211	120120	132132	173193	162166	117119	141141	134137	96100
PR 3	PR3-F3	MA	26997	9494	275275	9494	123123	142175	9797	207211	120120	132132	173193	162166	117119	141141	134137	96100
PR 3	PR3-F4	MA	26997	9494	275275	9494	123123	142175	9797	207211	120120	132132	173193	162166	117119	141141	134137	96100
PR 3	PR3-F5	MA	26997	9496	275275	9494	123123	136187	9797	205207	120120	132135	169193	158166	111111	143145	134137	9698
PR 4	PR4-F1	MA	6393	9496	275275	9494	123123	136175	9797	205211	118118	132135	169197	162162	115115	141141	137137	9698
PR 4	PR4-F2	MA	6393	9496	275275	9494	123123	136175	9797	205211	118118	132135	169197	162162	115115	141141	137137	9698
PR 4	PR4-F3	MA	6393	9496	275275	9494	123123	136175	9797	205211	118118	132135	169197	162162	115115	141141	137137	9698
PR 4	PR4-F4	MA	6393	9496	275275	9494	123123	136175	9797	205211	118118	132135	169197	162162	115115	141141	137137	9698
PR 4	PR4-F5	MA	6393	9496	275275	9494	123123	136175	9797	205211	118118	132135	169197	162162	115115	141141	137137	9698
PR 5	PR5-F1	MA	6332	9494	275275	9494	123123	139175	9797	205205	120120	135135	169197	158162	115115	141141	134134	9698
PR 5	PR5-F2	MA	6332	9494	275275	9494	123123	139175	9797	205205	120120	135135	169197	158162	115115	141141	134134	9698

PR 5	PR5-F3	MA	6332	9494	275275	9494	123123	139175	9797	205205	120120	135135	169197	158162	115115	141141	134134	9698
PR 5	PR5-F4	MA	6332	9494	275275	9494	123123	139175	9797	205205	120120	135135	169197	158162	115115	141141	134134	9698
PR 5	PR5-F5	MA	6332	9494	275275	9494	123123	139139	9797	205205	120120	135135	169197	158162	115115	141141	134134	9698
PR 6	PR6-F1	MA	1359	9494	275275	9494	123123	139175	9797	205205	120120	135135	169197	158162	115115	141141	134134	9698
PR 6	PR6-F2	MA	1359	9494	275275	9494	123123	139175	9797	205205	120120	135135	169197	158162	115115	141141	134134	9698
PR 6	PR6-F3	MA	1359	9494	275275	9494	123123	139175	9797	205205	120120	135135	169197	158162	115115	141141	134134	9698
PR 6	PR6-F4	MA	1359	9494	275275	9494	123123	139175	9797	205205	120120	135135	169197	158162	115115	141141	134134	9698
PR 6	PR6-F5	MA	1359	9494	275275	9494	123123	139175	9797	205205	120120	135135	169197	158162	115115	141141	134134	9698
PR 7	PR7-F1	MA	2037	9494	275275	94100	123123	139142	9797	203207	120120	132132	169169	162166	113115	141143	137137	9698
PR 7	PR7-F2	MA	2037	9494	275275	94100	123123	139142	9797	203205	120120	132132	169193	162166	115117	141143	137137	9698
PR 7	PR7-F3	MA	2037	9494	275275	94100	123123	139142	9797	203205	120120	132132	169193	162166	113115	141143	137137	9698
PR 7	PR7-F4	MA	2037	9494	275275	94100	123123	139142	9797	203205	120120	132132	169193	162166	113115	141143	137137	9698
PR 7	PR7-F5	MA	2037	9494	275275	94100	123123	139142	9797	203205	120120	132132	169193	162166	113115	141143	137137	9698
PR 8	PR8-F1	MA	22508	9494	275275	9494	123123	172172	9797	205205	120120	135135	173193	158162	125125	141143	134134	9494
PR 8	PR8-F2	MA	22508	9494	275275	9494	123123	172172	9797	205205	120120	135135	173193	158162	125125	141143	134134	9494
PR 8	PR8-F3	MA	22508	9494	275275	9494	123123	172172	9797	205205	120120	135135	173193	158162	125125	141143	134134	9494
PR 8	PR8-F4	MA	22508	9494	275275	9494	123123	172172	9797	205205	120120	135135	173193	158162	125125	141143	134134	9494
PR 8	PR8-F5	MA	22508	9494	275275	9494	123123	172172	9797	205205	120120	135135	173193	158162	125125	141143	134134	9494
PR 9	PR9-F1	MA	8249	9494	275283	9494	123123	136139	9797	205205	120120	135135	173193	162166	105119	141143	134143	9698
PR 9	PR9-F2	MA	8249	9494	275283	9494	123129	166175	9797	203207	---	132135	169173	158162	105119	141145	134134	96100
PR 9	PR9-F3	MA	8249	9494	275283	9494	123129	166175	9797	203207	---	132135	169173	158162	105119	141145	134134	96100
PR 9	PR9-F4	MA	8249	9494	275283	9494	123129	166175	9797	203207	124124	132135	169173	158162	105119	141145	134134	96100
PR 9	PR9-F5	MA	8249	9494	275283	9494	123129	166175	9797	203207	---	132135	169173	158162	105119	141145	134134	96100
PR 10	PR10-F1	MA	6195	9496	275275	9494	123123	136178	9797	203205	---	135135	193197	166166	107115	145145	134137	9696
PR 10	PR10-F2	MA	6195	9496	275275	9494	123123	136178	9797	203205	---	135135	193197	166166	107115	145145	134137	9696
PR 10	PR10-F3	MA	6195	9496	275275	9494	123123	136178	9797	203205	---	135135	193197	166166	107115	145145	134137	9696
PR 10	PR10-F4	MA	6195	9496	275275	9494	123123	136178	9797	203205	---	135135	193197	166166	107115	145145	134137	9696

PR 10	PR10-F5	MA	6195	9496	275275	9494	123123	136178	9797	203205	---	135135	193197	166166	107115	145145	134137	9696
FR 1	FR1-F1	MA	3876	9494	275275	100100	138138	151169	118118	203205	120120	135135	193193	162166	123127	139141	134137	9698
FR 1	FR1-F2	MA	3876	9494	275275	100100	138138	151169	118118	203205	120120	135135	193193	162166	123127	139141	134137	9698
FR 1	FR1-F3	MA	3876	9494	275275	100100	138138	151169	118118	203205	120120	135135	193193	162166	123127	139141	134137	9698
FR 1	FR1-F4	MA	3876	9494	275275	100100	138138	151169	118118	203205	120120	135135	193193	162166	123127	139141	134137	9698
FR 1	FR1-F5	MA	3876	9494	275275	100100	138138	151169	118118	203205	120120	135135	193193	162166	123127	139141	134137	9698
FR 2	FR2-F1	MA	5079	9494	275275	---	123123	---	9797	205205	---	132135	169193	162162	111123	139149	134134	9698
FR 2	FR2-F2	MA	5079	9494	275275	---	123123	136172	9797	205205	---	132135	169193	162162	111123	139149	134134	9698
FR 2	FR2-F3	MA	5079	9494	275275	---	123123	136172	9797	205205	---	132135	169193	162162	111123	139149	134134	9698
FR 2	FR2-F4	MA	5079	9494	275275	---	123123	136172	9797	205205	---	132135	169193	162162	111123	139149	134134	9698
FR 2	FR2-F5	MA	5079	9494	275275	---	123123	136172	9797	205205	---	132135	169193	162162	111123	139149	134134	9698
FR 3	FR3-F1	MA	14011	9494	275275	---	123123	136172	9797	205205	---	132135	169193	162162	111123	139149	134134	9698
FR 3	FR3-F2	MA	14011	9494	275275	---	123123	136172	9797	205205	---	132135	169193	162162	111123	139149	134134	9698
FR 3	FR3-F3	MA	14011	9494	275275	---	123123	136172	9797	205205	---	132135	169193	162162	111123	139149	134134	9698
FR 3	FR3-F4	MA	14011	9494	275275	---	123123	136172	9797	205205	---	132135	169193	162162	111123	139149	134134	9698
FR 3	FR3-F5	MA	14011	9494	275275	---	123123	136172	9797	205205	---	132135	169193	162162	111123	139149	134134	9698
FR 4	FR4-F1	MA	7079	9494	275275	9494	123129	172172	---	203205	120120	132132	193193	162162	123123	139139	134134	9696
FR 4	FR4-F2	MA	7079	9494	275275	9494	123129	172172	---	203205	120120	132132	193193	162162	119119	139139	134134	9696
FR 4	FR4-F3	MA	7079	9494	275275	9494	123123	139160	9797	203205	120120	132132	193193	162162	119119	139139	134134	9696
FR 4	FR4-F4	MA	7079	9494	275275	9494	123123	139160	9797	203205	120120	132132	193193	162162	119119	139139	134134	9696
FR 4	FR4-F5	MA	7079	9494	275275	9494	123129	172172	---	203205	120120	132132	193193	162162	119119	139139	134134	9696
FR 5	FR5-F1	MA	6551	9696	275275	94100	123123	160178	9797	205205	---	132135	169173	162166	95123	139143	134134	9698
FR 5	FR5-F2	MA	6551	9696	275275	94100	123123	160178	9797	205205	---	132135	169173	162166	95123	139143	134134	9698
FR 5	FR5-F3	MA	6551	9696	275275	94100	123123	160178	9797	205205	---	132135	169173	162166	95123	139143	134134	9698
FR 5	FR5-F4	MA	6551	9696	275275	94100	123123	160178	9797	205205	---	132135	169173	162166	95123	139143	134134	9698
FR 5	FR5-F5	MA	6551	9696	275275	94100	123123	160178	9797	205205	---	132135	169173	162166	95123	139143	134134	9698
FR 6	FR6-F1	MA	4599	9494	275275	---	123135	160184	9797	205205	120120	132132	169193	162166	123127	143143	140140	112112

FR 6	FR6-F2	MA	4599	9494	275275	---	123135	160184	9797	205205	120120	132132	169193	162166	123127	143143	140140	112112
FR 6	FR6-F3	MA	4599	9494	275275	---	123135	160184	9797	205205	120120	132132	169193	162166	123127	143143	140140	112112
FR 6	FR6-F4	MA	4599	9494	275275	---	123135	160184	9797	205205	120120	132132	169193	162166	123127	143143	140140	112112
FR 6	FR6-F5	MA	4599	9494	275275	---	123135	160184	9797	205205	120120	132132	169193	162166	123127	143143	140140	112112
FR 7	FR7-F1	MA	6909	9494	275279	9494	129129	136172	9797	207207	---	132135	169197	162162	119123	139143	134134	9698
FR 7	FR7-F2	MA	6909	9494	275279	9494	129129	136172	9797	207207	---	132135	169197	162162	119123	139143	134134	9698
FR 7	FR7-F3	MA	6909	9494	275279	9494	129129	136172	9797	207207	---	132135	169197	162162	119123	139143	134134	9698
FR 7	FR7-F4	MA	6909	9494	275279	9494	129129	136172	9797	207207	---	132135	169197	162162	119123	139143	134134	9698
FR 7	FR7-F5	MA	6909	9494	275279	9494	129129	136172	9797	207207	---	132135	169197	162162	119123	139143	134134	9698
FR 8	FR8-F1	MA	16239	9494	275283	9494	123129	136160	9797	205207	120120	132135	169193	162166	115115	141141	140140	9698
FR 8	FR8-F2	MA	16239	9494	275283	9494	123129	136160	9797	205207	120120	132135	169193	162166	115115	141141	140140	9698
FR 8	FR8-F3	MA	16239	9494	275283	9494	123129	136160	9797	205207	120120	132135	169193	162166	115115	141141	140140	9698
FR 8	FR8-F4	MA	16239	9494	275283	9494	123129	136160	9797	205207	120120	132135	169193	162166	115115	141141	140140	9698
FR 8	FR8-F5	MA	16239	9494	275283	9494	123129	136160	9797	205207	120120	132135	169193	162166	115115	141141	140140	9698
FR 9	FR9-F1	MA	13630	9494	275275	---	123123	184187	9797	205205	---	132135	193193	162162	111115	---	134134	9696
FR 9	FR9-F2	MA	13630	9494	275275	---	123123	184187	9797	205205	---	132135	173193	162162	111115	141143	134134	9696
FR 9	FR9-F3	MA	13630	9494	275275	---	123123	184187	9797	205205	---	132135	173193	162162	111115	141143	134134	9696
FR 9	FR9-F4	MA	13630	9494	275275	---	123123	184187	9797	205205	---	132135	173193	162162	111115	141143	134134	9696
FR 9	FR9-F5	MA	13630	9494	275275	---	123123	184187	9797	205205	---	132135	173193	162162	111115	141143	134134	9696
FR 10	FR10-F1	MA	11783	9496	275307	---	123135	136184	9797	205207	120120	132135	193197	162162	103111	139139	134137	9696
FR 10	FR10-F2	MA	11783	9496	275307	---	123135	136184	9797	205207	120120	132135	193197	162162	103111	139139	134137	9696
FR 10	FR10-F3	MA	11783	9496	275307	---	123135	136184	9797	205207	120120	132135	193197	162162	103111	139139	134137	9696
FR 10	FR10-F4	MA	11783	9496	275307	---	123135	136184	9797	205207	120120	132135	193197	162162	103111	139139	134137	9696
FR 10	FR10-F5	MA	11783	9496	275307	---	123135	136184	9797	205207	120120	132135	193197	162162	103111	139139	134137	9696
BR 1	BR1-F1	MA	12854	9496	275307	9494	123123	169172	9797	203205	114114	135135	193193	162166	111123	139143	134137	9698
BR 1	BR1-F2	MA	12854	9496	275307	9494	123123	169172	9797	203205	114114	135135	193193	162166	111123	139143	134137	9698
BR 1	BR1-F3	MA	12854	9496	275307	9494	123123	169172	9797	203205	114114	135135	193193	162166	111123	139143	134137	9698

BR 1	BR1-F4	MA	12854	9496	275307	9494	123123	169172	9797	203205	114114	135135	193193	162166	111123	139143	134137	9698
BR 1	BR1-F5	MA	12854	9496	275307	9494	123123	169172	9797	203205	114114	135135	193193	162166	111123	139143	134137	9698
BR 2	BR2-F1	ST	3164	9494	343343	9494	123123	139169	---	205207	---	135135	193193	162162	113113	141143	134134	9698
BR 2	BR2-F2	ST	3164	9494	343343	9494	123123	139169	---	205207	---	135135	193193	162162	113113	141143	134134	9698
BR 2	BR2-F3	ST	3164	9494	343343	9494	123123	139169	---	205207	---	135135	193193	162162	113113	141143	134134	9698
BR 2	BR2-F4	ST	3164	9494	343343	9494	123123	139169	---	205207	---	135135	193193	162162	113113	141143	134134	9698
BR 2	BR2-F5	ST	3164	9494	343343	9494	123123	139169	---	205207	---	135135	193193	162162	113113	141143	134134	9698
BR 3	BR3-F1	ST	4080	9696	275331	---	123129	136139	9797	205211	---	132135	193197	158166	111123	141143	134137	9696
BR 3	BR3-F2	ST	4080	9696	275331	---	123129	136139	9797	205211	---	132135	193197	158166	111123	141143	134137	9696
BR 3	BR3-F3	ST	4080	9696	275331	---	123129	136139	9797	205211	---	132135	193197	158166	111123	141143	134137	9696
BR 3	BR3-F4	ST	4080	9696	275331	---	123129	136139	9797	205211	---	132135	193197	158166	111123	141143	134137	9696
BR 3	BR3-F5	ST	4080	9696	275331	---	123129	136139	9797	205211	---	132135	193197	158166	111123	141143	134137	9696
BR 4	BR4-F1	ST	3591	9696	275278	9494	123123	142175	9797	203207	120120	132135	193193	162162	111115	139141	134137	9698
BR 4	BR4-F2	ST	3591	9696	275278	9494	123123	142175	9797	203207	120120	132135	193193	162162	111115	139141	134137	9698
BR 4	BR4-F3	ST	3591	9696	275278	9494	123123	142175	9797	203207	120120	132135	193193	162162	111115	139141	134137	9698
BR 4	BR4-F4	ST	3591	9696	275278	9494	123123	142175	9797	203207	120120	132135	193193	162162	111115	139141	134137	9698
BR 4	BR4-F5	ST	3591	9696	275278	9494	123123	142175	9797	203207	120120	132135	193193	162162	111115	139141	134137	9698
BR 5	BR5-F1	ST	1457	9496	283283	9494	123129	136136	9797	205207	120120	132132	169169	158162	101113	139141	134134	9696
BR 5	BR5-F2	ST	1457	9496	283283	9494	123129	136136	9797	205207	120120	132132	169169	158162	101113	139141	134134	9696
BR 5	BR5-F3	ST	1457	9496	283283	9494	123129	136136	9797	205207	120120	132132	169169	158162	101113	139141	134134	9696
BR 5	BR5-F4	ST	1457	9496	283283	9494	123129	136136	9797	205207	120120	132132	169169	158162	101113	139141	134134	9696
BR 5	BR5-F5	ST	1457	9496	283283	9494	123129	136136	9797	205207	120120	132132	169169	158162	101113	139141	134134	9696
BR 6	BR6-F1	ST	4004	9494	283283	9494	123123	163178	9797	205205	---	135135	193197	162166	115119	141143	137137	9696
BR 6	BR6-F2	ST	4004	9494	283283	9494	123123	163178	9797	205205	---	135135	193197	162166	115119	141143	137137	9696
BR 6	BR6-F3	ST	4004	9494	283283	9494	123123	163178	9797	205205	---	135135	193197	162166	115119	141143	137137	9696
BR 6	BR6-F4	ST	4004	9494	283283	9494	123123	163178	9797	205205	---	135135	193197	162166	115119	141143	137137	9696
BR 6	BR6-F5	ST	4004	9494	283283	9494	123123	163178	9797	205205	---	135135	193197	162166	115119	141143	137137	9696

BR 7	BR7-F1	MA	2047	9496	283283	9494	123129	136136	9797	205207	120120	132132	169169	158162	101113	139141	134134	9696
BR 7	BR7-F2	MA	2047	9496	283283	9494	123129	136136	9797	205207	120120	132132	169169	158162	101113	139141	134134	9696
BR 7	BR7-F3	MA	2047	9496	283283	9494	123129	136136	9797	205207	120120	132132	169169	158162	101113	139141	134134	9696
BR 7	BR7-F4	MA	2047	9496	283283	9494	123129	136136	9797	205207	120120	132132	169169	158162	101113	139141	134134	9696
BR 7	BR7-F5	MA	2047	9496	283283	9494	123129	136136	9797	205207	120120	132132	169169	158162	101113	139141	134134	9696
BR 8	BR8-F1	ST	3111	9496	275283	9494	123123	139184	9797	203205	---	135135	197197	162166	93123	139139	134134	94100
BR 8	BR8-F2	ST	3111	9496	275283	9494	123123	139184	9797	203205	---	135135	197197	162166	93123	139139	134134	94100
BR 8	BR8-F3	ST	3111	9496	275283	9494	123123	139184	9797	203205	---	135135	197197	162166	93123	139139	134134	94100
BR 8	BR8-F4	ST	3111	9496	275283	9494	123123	139184	9797	203205	---	135135	197197	162166	93123	139139	134134	94100
BR 8	BR8-F5	ST	3111	9496	275283	9494	123123	139184	9797	203205	---	135135	197197	162166	93123	139139	134134	94100
UP 1	UP1-F1	EN	49862	9494	275307	100100	123123	172175	9797	205205	120120	132135	197197	162162	113125	139143	134137	9698
UP 1	UP1-F2	EN	49862	9494	275307	100100	123123	172175	9797	205205	120120	132135	197197	162162	113125	139143	134137	9698
UP 1	UP1-F3	EN	49862	9494	275307	100100	123123	172175	9797	205205	120120	132135	197197	162162	113125	139143	134137	9698
UP 1	UP1-F4	EN	49862	9494	275307	100100	123123	172175	9797	205205	120120	132135	197197	162162	113125	139143	134137	9698
UP 1	UP1-F5	EN	49862	9494	275307	100100	123123	172175	9797	205205	120120	132135	197197	162162	113125	139143	134137	9698
UP 2	UP2-F1	ST	63523	9496	275275	9494	123123	136172	9797	205207	---	135135	169193	166166	113119	139139	137137	9698
UP 2	UP2-F2	ST	63523	9496	275275	9494	123123	136172	9797	205207	---	135135	169193	166166	113119	139139	137137	9698
UP 2	UP2-F3	ST	63523	9496	275275	9494	123123	136172	9797	205207	---	135135	169193	166166	113119	139139	137137	9698
UP 2	UP2-F4	ST	63523	9496	275275	9494	123123	136172	9797	205207	---	135135	169193	166166	113119	139139	137137	9698
UP 2	UP2-F5	ST	63523	9496	275275	9494	123123	136172	9797	205207	---	135135	169193	166166	113119	139139	137137	9696
UP 3	UP3-F1	ST	7318	9494	275275	---	123129	163184	9797	207207	114120	132135	193193	162162	117123	139141	134134	9494
UP 3	UP3-F2	ST	7318	9494	275275	---	123129	163184	9797	207207	---	132135	193193	162162	117123	139141	134134	9494
UP 3	UP3-F3	ST	7318	9494	275275	---	123129	163184	9797	207207	---	132135	193193	162162	117123	139141	134134	9494
UP 3	UP3-F4	ST	7318	9494	275275	---	123129	163184	9797	207207	---	132135	193193	162162	117123	139141	134134	9494
UP 3	UP3-F5	ST	7318	9494	275275	---	123129	163184	9797	207207	---	132135	193193	162162	117123	139141	134134	9494
UP 4	UP4-F1	ST	35400	9494	275283	---	123123	139160	9797	205207	---	132135	193197	162162	115119	141143	134134	9696
UP 4	UP4-F2	ST	35400	9494	275283	---	123123	139160	9797	205207	---	132135	193197	162162	115119	141143	134134	9696

UP 4	UP4-F3	ST	35400	9494	275283	---	123123	139160	9797	205207	---	132135	193197	162162	115119	141143	134134	9696
UP 4	UP4-F4	ST	35400	9494	275283	---	123123	139160	9797	205207	---	132135	193197	162162	115119	141143	134134	9696
UP 4	UP4-F5	ST	35400	9494	275283	---	123123	139160	9797	205207	---	132135	193197	162162	115119	141143	134134	9696
UP 5	UP5-F1	ST	29500	9494	275283	9494	123135	160169	9797	205205	114120	132132	193197	162162	111119	139139	134134	9696
UP 5	UP5-F2	ST	29500	9494	275283	9494	123135	160169	9797	205205	114120	132132	193197	162162	111119	139139	134134	9696
UP 5	UP5-F3	ST	29500	9494	275283	9494	123135	160169	9797	205205	114120	132132	193197	162162	111119	139139	134134	9696
UP 5	UP5-F4	ST	29500	9494	275283	9494	123135	160169	9797	205205	114120	132132	193197	162162	111119	139139	134134	9696
UP 6	UP6-F1	ST	42448	9494	275275	100100	123129	142178	9797	205207	120120	135135	169169	158162	115115	139139	134137	9696
UP 6	UP6-F2	ST	42448	9494	275275	100100	123129	142178	9797	205207	120120	135135	169169	158162	115115	139139	134137	9696
UP 6	UP6-F3	ST	42448	9494	275275	100100	123129	142178	9797	205207	120120	135135	169169	158162	111115	139139	134137	9696
UP 6	UP6-F4	ST	42448	9494	275275	100100	123129	142178	9797	205207	120120	135135	169169	158162	111115	139139	134137	9696
UP 6	UP6-F5	ST	42448	9494	275275	100100	123129	142178	9797	205207	120120	135135	169169	158162	111115	139139	134137	9696
UP 7	UP7-F1	EN	70471	9494	275275	---	123123	160184	9797	203205	---	132135	169197	162166	117125	139143	134137	9696
UP 7	UP7-F2	EN	70471	9494	275275	---	123123	160184	9797	203205	---	132135	169197	162166	117125	139143	134137	9696
UP 7	UP7-F3	EN	70471	9494	275275	---	123123	160184	9797	203205	---	132135	169197	162166	117125	139143	134137	9696
UP 7	UP7-F4	EN	70471	9494	275275	---	123123	160184	9797	203205	---	132135	169197	162166	117125	139143	134137	9696
UP 7	UP7-F5	EN	70471	9494	275275	---	123123	160184	9797	203205	---	132135	169197	162166	117125	139143	134137	9696
UP 8	UP8-F1	ST	31470	9496	275307	---	129129	169184	9797	203207	120120	132135	193193	162162	113119	139141	134134	9698
UP 8	UP8-F2	ST	31470	9496	275307	---	129129	169184	9797	203207	120120	132135	193193	162162	113119	139141	134134	9698
UP 8	UP8-F3	ST	31470	9496	275307	---	129129	169184	9797	203207	120120	132135	193193	162162	113119	139141	134134	9698
UP 8	UP8-F4	ST	31470	9496	275307	---	129129	169184	9797	203207	120120	132135	193193	162162	113119	139141	134134	9698
UP 8	UP8-F5	ST	31470	9496	275307	---	129129	169184	9797	203207	120120	132135	193193	162162	113119	139141	134134	9698
UP 9	UP9-F1	ST	21123	9494	275275	9494	123129	136139	9797	205205	---	132135	169193	162162	9597	139139	134137	112114
UP 9	UP9-F2	ST	21123	9494	275275	9494	123129	136139	9797	205205	---	132135	169193	162162	9597	139139	134137	112114
UP 9	UP9-F3	ST	21123	9494	275275	9494	123129	136139	9797	205205	---	132135	169193	162162	117123	139139	134137	9698
UP 9	UP9-F4	ST	21123	9494	275275	9494	123129	136139	9797	205205	---	132135	169193	162162	117123	139139	134137	9698
UP 9	UP9-F5	ST	21123	9494	275275	9494	123129	136139	9797	205205	---	132135	169193	162162	117123	139139	134137	9698

UP 10	UP10-F1	ST	28166	9696	275283	9494	123135	172181	9797	203205	120120	132135	173193	162162	111115	141143	134134	94100
UP 10	UP10-F2	ST	28166	9696	275283	9494	123135	172181	9797	203205	120120	132135	173193	162162	111115	141143	134134	94100
UP 10	UP10-F3	ST	28166	9696	275283	9494	123135	172181	9797	203205	120120	132135	173193	162162	111115	141143	134134	94100
UP 10	UP10-F4	ST	28166	9696	275283	9494	123135	172181	9797	203205	120120	132135	173193	162162	111115	141143	134134	94100
UP 10	UP10-F5	ST	28166	9696	275283	9494	123135	172181	9797	203205	120120	132135	173193	162162	111115	141143	134134	94100
UP 11	UP11-F1	ST	984	9496	275275	9494	123129	136163	9797	205207	---	132132	193193	162162	93115	141141	134137	9496
UP 11	UP11-F2	ST	984	9496	275275	9494	123129	136163	9797	205207	---	132132	193193	162162	93115	141141	134137	9496
UP 11	UP11-F3	ST	984	9496	275275	9494	123129	136163	9797	205207	---	132132	193193	162162	93115	141141	134137	9496
UP 11	UP11-F4	ST	984	9496	275275	9494	123129	136163	9797	205207	---	132132	193193	162162	93115	141141	134137	9496
UP 11	UP11-F5	ST	984	9496	275275	9494	123129	136163	9797	205207	120120	132135	173193	162166	93115	141141	134137	9496
UP 12	UP12-F1	ST	5410	9496	275275	9494	123129	136163	9797	205207	---	132132	193193	162162	93115	141141	134137	9496
UP 12	UP12-F2	ST	5410	9496	275275	94100	123129	136163	9797	205207	120120	132135	173193	162166	93115	141141	134137	9496
UP 12	UP12-F3	ST	5410	9496	275275	94100	123129	136163	9797	205207	120120	132135	173193	162166	93115	141141	134137	9496
UP 12	UP12-F4	ST	5410	9496	275275	9494	123129	136163	9797	205207	---	132132	193193	162162	93115	141141	134137	9496
UP 12	UP12-F5	ST	5410	9496	275275	9494	123129	136163	9797	205207	---	132132	193193	162162	93115	141141	134137	9496
UP 13	UP13-F1	ST	817	9496	275275	9494	123129	136163	9797	205207	---	132132	193193	162162	93115	141141	134137	9496
UP 13	UP13-F2	ST	817	9496	275275	9494	123129	136163	9797	205207	120120	132132	193193	162162	93115	141141	134137	9496
UP 13	UP13-F3	ST	817	9496	275275	94100	123129	136163	9797	205207	120120	132135	173193	162166	93115	141141	134137	9496
UP 13	UP13-F4	ST	817	9496	275275	9494	123129	136163	9797	205207	120120	132132	193193	162166	93115	141141	134137	9496
UP 13	UP13-F5	ST	817	9496	275275	9494	123129	136163	9797	205207	---	132132	193193	162162	93115	141141	134137	9496
MD 1	MD1-F1	EN	16187	9496	275283	9494	123135	139139	9797	205205	---	135135	169193	158162	111113	141143	137137	9696
MD 1	MD1-F2	EN	16187	9496	275283	9494	123135	139139	9797	205205	---	135135	169193	158162	111113	141143	137137	9696
MD 1	MD1-F3	EN	16187	9496	275283	9494	123135	139139	9797	205205	---	135135	169193	158162	111113	141143	137137	9696
MD 1	MD1-F4	EN	16187	9496	275283	9494	123135	139139	9797	205205	---	135135	169193	158162	111113	141143	137137	9696
MD 1	MD1-F5	EN	16187	9496	275283	9494	123135	139139	9797	205205	---	135135	169193	158162	111113	141143	137137	9696
MD 2	MD2-F1	ST	25031	9494	275275	---	129135	136139	9797	203207	---	135135	173193	158162	103111	139141	134137	9698
MD 2	MD2-F2	ST	25031	9494	275275	---	129135	136139	9797	203207	---	135135	173193	158162	103111	139141	134137	9698

MD 2	MD2-F3	ST	25031	9494	275275	---	129135	136139	9797	203207	---	135135	173193	158162	103111	139141	134137	9698
MD 2	MD2-F4	ST	25031	9494	275275	---	129135	136139	9797	203207	---	135135	173193	158162	103111	139141	134137	9698
MD 2	MD2-F5	ST	25031	9494	275275	---	129135	136139	9797	203207	---	135135	173193	158162	103111	139141	134137	9698
MD 3	MD3-F1	ST	13571	9496	275275	100100	129129	160169	---	205205	120120	135135	193197	162162	119127	139143	134134	9494
MD 3	MD3-F2	ST	13571	9496	275275	100100	129129	160169	---	205205	120120	135135	193197	162162	119127	139143	134134	9494
MD 3	MD3-F3	ST	13571	9496	275275	100100	129129	160169	---	205205	120120	135135	193197	162162	119127	139143	134134	9494
MD 3	MD3-F4	ST	13571	9496	275275	100100	129129	160169	---	205205	120120	135135	193197	162162	119127	139143	134134	9494
MD 3	MD3-F5	ST	13571	9496	275275	100100	129129	160169	---	205205	120120	135135	193197	162162	119127	139143	134134	9494
MD 4	MD4-F1	MA	22182	9494	283331	9494	123123	175175	9797	207211	---	135135	193193	162162	111123	141143	134137	9696
MD 4	MD4-F2	MA	22182	9494	283331	9494	123123	175175	9797	207211	---	135135	193193	162162	111123	141143	134137	9696
MD 4	MD4-F3	MA	22182	9494	283331	9494	123123	175175	9797	207211	---	135135	193193	162162	111123	141143	134137	9696
MD 4	MD4-F4	MA	22182	9494	275283	9494	123129	175184	9797	211211	124124	135135	193193	162162	111111	---	137137	9696
MD 4	MD4-F5	MA	22182	9494	283331	9494	123123	175175	9797	207211	---	135135	193193	162162	111123	141143	134137	9696
MD 5	MD5-F1	EN	10415	9496	275275	---	123129	163169	---	205205	120120	135135	169173	162162	119123	139143	134134	9696
MD 5	MD5-F2	EN	10415	9496	275275	---	123129	163169	---	205205	120120	135135	169173	162162	119123	139143	134134	9696
MD 5	MD5-F3	EN	10415	9496	275275	---	123129	163169	---	205205	120120	135135	169173	162162	119123	139143	134134	9696
MD 5	MD5-F4	EN	10415	9494	275275	---	123129	163169	---	203205	120120	135135	169169	150150	119123	139143	134134	9698
MD 5	MD5-F5	EN	10415	9496	275275	---	123129	163169	---	205205	120120	135135	169173	162162	119123	139143	134134	9696
MD 6	MD6-F1	EN	13638	9496	275307	9494	123123	169172	---	203207	114118	132135	193193	162166	103111	139143	134134	9696
MD 6	MD6-F2	EN	13638	9496	275307	9494	123123	169172	---	203207	114118	132135	193193	162166	103111	139143	134134	9696
MD 6	MD6-F3	EN	13638	9496	275307	9494	123123	169172	---	203207	114118	132135	193193	162166	103111	139143	134134	9696
MD 6	MD6-F4	EN	13638	9496	275307	9494	123123	169172	---	203207	114118	132135	193193	162166	103111	139143	134134	9696
MD 6	MD6-F5	EN	13638	9496	275307	9494	123123	169172	---	203207	114118	132135	193193	162166	103111	139143	134134	9696
MD 7	MD7-F1	EN	3282	9494	275275	---	123123	142142	9797	207211	---	132135	169193	162166	93111	139143	134137	9698
MD 7	MD7-F2	EN	3282	9494	275275	---	123123	142142	9797	207211	---	132135	169193	162166	93111	139143	134137	9698
MD 7	MD7-F3	EN	3282	9494	275275	---	123123	142142	9797	207211	---	132135	169193	162166	93111	139143	134137	9698
MD 7	MD7-F4	EN	3282	9494	275275	---	123123	142142	9797	207211	---	132135	169193	162166	93111	139143	134137	9698

MD 7	MD7-F5	EN	3282	9494	275275	---	123123	142142	9797	207211	---	132135	169193	162166	93111	139143	134137	9698
MD 8	MD8-F1	MA	39802	9496	275275	9494	123123	169169	---	205207	120120	132132	173197	162166	91123	139139	134137	9698
MD 8	MD8-F2	MA	39802	9496	275275	9494	123123	169169	---	205207	120120	132132	173197	162166	91123	139139	134137	9698
MD 8	MD8-F3	MA	39802	9496	275275	9494	123123	169169	---	205207	120120	132132	173197	162166	91123	139139	134137	9698
MD 8	MD8-F4	MA	39802	9496	275275	9494	123123	169169	---	205207	120120	132132	173197	162166	91123	139139	134137	9698
MD 8	MD8-F5	MA	39802	9496	275275	9494	123123	169169	---	205207	120120	132132	173197	162166	91123	139139	134137	9698
MD 9	MD9-F1	ST	30042	9494	275275	---	123123	178184	9797	207211	---	132135	173193	162162	93119	139143	134134	9696
MD 9	MD9-F2	ST	30042	9494	275275	---	123123	178184	9797	207211	---	132135	173193	162162	93119	139143	134134	9696
MD 9	MD9-F3	ST	30042	9494	275275	---	123123	178184	9797	207211	---	132135	173193	162162	93119	139143	134134	9696
MD 9	MD9-F4	ST	30042	9494	275275	---	123123	178184	9797	207211	---	132135	173193	162162	93119	139143	134134	9696
MD 9	MD9-F5	ST	30042	9494	275275	---	123123	178184	9797	207211	---	132135	173193	162162	93119	139143	134134	9696
MD 10	MD10-F1	MA	10448	9696	275275	94100	123123	175184	9797	205207	120120	132135	193193	162162	93119	139141	134134	9698
MD 10	MD10-F2	MA	10448	9696	275275	94100	123123	175184	9797	205207	120120	132135	193193	162162	93119	139141	134134	9698
MD 10	MD10-F3	MA	10448	9696	275275	94100	123123	175184	9797	205207	120120	132135	193193	162162	93119	139141	134134	9698
MD 10	MD10-F4	MA	10448	9696	275275	94100	123123	175184	9797	205207	120120	132135	193193	162162	93119	139141	134134	9698
MD 10	MD10-F5	MA	10448	9696	275275	94100	123123	175184	9797	205207	120120	132135	193193	162162	93119	139141	134134	9698

ID Col, Identification of the colony; ID Frag, Identification of each fragment within a colony; Mor, Colony morphology and Size, Colony size. Habitat: PR, Patch reef; FR, Fringing reef; BR, Back reef; UP, Upper slope and MD, Mid slope. Morphology: MA, Massive; EN, Encrusting and ST, Sheet tree. --- No amplification (3 repetitions). Grey squares indicate allelic variation for a given locus among the five fragments collected from each of the 51 sampled colonies.
